# Supplementary material for: SyNDI: synchronous network data integration framework
Source: BMC Bioinformatics. 2018 Nov 6;19:403. doi: 10.1186/s12859-018-2426-5 (PMC6219086; doi:10.1186/s12859-018-2426-5)
Supplement: Supplementary file 5 — Files of the example presented in Additional file 4. This additional file is a zip package that contains all files used in Additional file 4. Detailed descriptions of each of these files are presented in a README file included in this zip package. (GZ 30411 kb) [file 12859_2018_2426_MOESM5_ESM.gz › SyNDITools/Meme2Fimo/meme_4.9.0_4.tar.gz!/meme_4.9.0/website/html/metameme-help-format.in]

Meta-MEME v3.3


> ### Sequence formats
>
> ---
>
> The preferred sequence format for Meta-MEME is Fasta format. For
> example,
>
> ```
> >ICYA_MANSE Insecticyanin A form blue biliprotein) 
> GDIFYPGYCPDVKPVNDFDLSAFAGAWHEIAKLPLENENQGKCTIAEYKYDGKK
> ASVYNSFVSNGVKEYMEGDLEIAPDA
> >LACB_BOVIN Beta-lactoglobulin precursor (BETA-LG)
> MKCLLLALALTCGAQALIVTQTMKGLDIQKVAGTWYSLAMAASDISLLDAQSAPL
> RVYVEELKPTPEGDLEILLQKW
> ```
>
> Sequences start with a header line followed by sequence lines. The
> header line begins with the character ">", followed by a unique
> name without any spaces, followed by (optional) descriptive text.
> After the header line come the actual sequence lines. Spaces and
> blank lines are ignored. Sequences may be in capital, lowercase or
> both.
>
> The web version of Meta-MEME also accepts protein and DNA
> sequences in any of the following formats by converting them to
> Fasta format.
>
> - IG/Stanford, used by Intelligenetics and others
> - GenBank/GB, genbank flatfile format
> - NBRF format
> - EMBL, EMBL flatfile format
> - DNAStrider, for common Mac program
> - Fitch format, limited use
> - Pearson/Fasta, a common format used by Fasta programs and
>   others
> - Zuker format, limited use
> - Olsen, format printed by Olsen VMS sequence editor
> - Phylip3.2, sequential format for Phylip programs
> - Phylip, interleaved format for Phylip programs (v3.3,
>   v3.4)
> - MSF multi sequence format used by GCG software
> - PAUP's multiple sequence (NEXUS) format
> - PIR/CODATA format used by PIR
> - ASN.1 format used by NCBI
>
> ---
>
> The Meta-MEME web site uses the 
> ReadSeq program to read in sequences. ReadSeq is copyright 1990
> by D. G. Gilbert, Biology Dept., Indiana University.
>
> ---
>
> Return to the Meta-MEME home page.
>
> *Please send comments and questions to:
> @METAMEME\_CONTACT@.*
